# Family Talk versus usual services in improving child and family psychosocial functioning in families with parental mental illness (PRIMERA—Promoting Research and Innovation in Mental hEalth seRvices for fAmilies and children): study protocol for a randomised controlled trial

**DOI:** 10.1186/s13063-021-05199-4

**Published:** 2021-04-01

**Authors:** Mairead Furlong, Sinead McGilloway, Christine Mulligan, Colm McGuinness, Nuala Whelan

**Affiliations:** 1 Centre for Mental Health and Community Research, Department of Psychology and Social Sciences Institute, Maynooth University, Maynooth, Co. Kildare Ireland; 2 Department of Business, Technological University Dublin, Blanchardstown Campus, Dublin, Ireland; 3 Department of Sociology, Maynooth University, Maynooth, Co. Kildare Ireland

**Keywords:** Mental illness, Children, Families, Family talk, Intergenerational transmission, Intervention, Parents, Prevention, Protocol, Randomised controlled trial

## Abstract

**Background:**

Parental mental illness is common and can lead to dependent children incurring a high risk of developing mental disorders, physical illness, and impaired educational and occupational outcomes. Family Talk is one of the better known interventions designed to prevent the intergenerational transmission of mental illness. However, its evidence base is small, with few robust independent randomised controlled trials, and no associated process or cost evaluations. The PRIMERA (Promoting Research and Innovation in Mental hEalth seRvices for fAmilies and children) research programme involves a mixed method evaluation of Family Talk which is being delivered in mental health settings in Ireland to improve child and family psychosocial functioning in families with parental mental illness.

**Methods:**

The study comprises a multi-centre, randomised controlled trial (RCT), with nested economic and process evaluations, to assess the clinical and cost-effectiveness and implementation mechanisms of Family Talk compared to usual services. The study is being conducted in 15 adult and child mental health settings in Ireland. Families with a parent with mental illness, and children aged 5–18 years (*n* = 144 families) will be randomised to either the 7-session Family Talk programme (*n* = 96) or to standard care (*n* = 48) using a 2:1 allocation ratio. The primary outcomes are child psychosocial functioning and family functioning. Secondary outcomes are as follows: understanding and experience of parental mental illness, parental mental health, child and parental resilience, partner wellbeing and service utilisation. Blind assessments will take place at pre-intervention and at 6- and 12-month follow-up.

**Discussion:**

Given the prevalence and burden of intergenerational mental illness, it is imperative that prevention through evidence-based interventions becomes a public health priority. The current study will provide an important contribution to the international evidence base for Family Talk whilst also helping to identify key implementation lessons in the scaling up of Family Talk, and other similar interventions, within routine mental health settings.

**Trial registration:**

ISRCTN Registry, ISRCTN13365858. Registered 5th February 2019.

**Supplementary Information:**

The online version contains supplementary material available at 10.1186/s13063-021-05199-4.

## Background

Approximately one in five adults worldwide is affected by mental illness, many of whom are parents [1]. It is estimated that 23% of all families have at least one parent who has or had a mental illness, with serious and far-reaching implications for child welfare and outcomes across the lifespan [2, 3]. In the Republic of Ireland (RoI), 20% of adults suffer from a mental illness—the third highest incidence across 36 countries in Europe—costing the Irish state €11 billion per year [4]. It is estimated that 280,000 children in the RoI are dependent on parents who have a mental illness [5]. In addition, studies indicate that 25 to 68% of adult mental health service users are parents, and 35 to 60% of children presenting at child and adolescent mental health services have a parent with mental illness [6, 7]. Worryingly, due to the historical and current segregation of adult and child mental health services in the RoI (and other jurisdictions), parenting status is commonly not recorded/investigated by mental health professionals, thereby compounding the vulnerability of these ‘hidden’ children [8, 9].

It is well documented that children of parents with mental illness (COPMI) are at elevated risk for a range of adverse outcomes, including infant mortality, developmental delay, attachment problems, abuse and neglect, medical illness, and various mental disorders (e.g. depression, anxiety, substance abuse, suicidal behaviour), as well as impairments to educational and occupational prospects [3, 10, 11]. These children are 3 to 13 times more likely to develop psychopathology than children who do not have a parent with a mental illness and are five times more likely to utilise health, social, and mental health services [3]. Furthermore, a large body of research provides evidence for both transgenerational *equifinality* and *multifinality*; that is, children are at a high risk of developing respectively, either the same diagnosis as their parent or a broad range of mental disorders [11–13]. Overall, longitudinal studies indicate that the life-time risk for developing a serious mental illness ranges from 41 to 77% for children of mentally ill parents, irrespective of their psychiatric diagnoses [3].

The intergenerational transmission of mental illness is currently considered to involve a complex interplay of genetic and prenatal factors, parent-child interactions, and family and environmental influences, such as social deprivation, family conflict, substance misuse, and lack of access to appropriate services [3]. The transmission of risk from parents to children, in particular, is significantly mediated by the impact of parental symptoms on parenting competence and parent-child interactions including, for example, parental withdrawal, neglect, rejection, hostility, and insensitive and erratic attunement [3]. COPMI typically report a lack of communication about, and understanding of, mental illness; for instance, they commonly fear that their parent will never recover/will die, that they too will inevitably develop a mental disorder, and that they are to blame for their parent’s condition. They also frequently undertake considerable caring duties, experience isolation, shame, stigma, and report impaired peer interactions and school engagement [14–16].

Given the prevalence and burden of parental mental illness, there has been a growing recognition of the need for more integrated and effective prevention and early-intervention approaches to protect children from developing mental health disorders [17]. In the last 15 years, a number of reviews have investigated a range of different interventions to prevent child mental illness (most typically depression), with promising, if not definitive, evidence of effectiveness [17–22]. Interventions which have been evaluated vary in terms of the person targeted (child, parent or family), group or individual format, length of intervention, and type of therapeutic modality and content (e.g. psycho-education, cognitive-behavioural strategies, parenting skills) [9, 19]. An important and rigorously conducted systematic review undertaken by Siegenthaler et al. (2012) included only 13 randomised controlled trials (*N* = 1490 children of parents with a mental illness) to assess the effectiveness of interventions designed to prevent mental illness in COPMI [21]. Overall, the results showed that the 13 interventions under investigation decreased the risk of developing mental illness for children by up to 40%, predominantly through parent or family-mediated programmes [21]. Notably, the Family Talk intervention—which was the focus of interest in 4 of the 13 studies—emerged as one of the more effective programmes [23–26].

Family Talk (FT) was developed by William Beardslee and colleagues in the USA in the 1980s and is a manualised, 7-session, strengths-based, psycho-educational, whole-family approach designed to enhance family communication and understanding of parental mental illness, improve family interpersonal relationships, and promote child resilience and utilisation of social supports [23]. In recent years, the programme has also been evaluated in Germany using a quasi-experimental design [27] and is currently being piloted in Chile [28] and Greece [29] using RCTs. Positive effects on child understanding of mental illness, child resilience, and internalising symptoms have been reported at post intervention and at 1.5- and 4.5-year follow-ups across the five FT evaluations [23–27]. Furthermore, there is evidence that FT may also promote the parent’s mental health recovery. For instance, diagnoses of parental affective disorders reduced from 90 to 66% at 4.5-year follow-up, and from 43 to 31% for non-affective disorders [25]. In addition, whilst originally designed to prevent the intergenerational transmission of depression, FT has been shown to be safe, feasible, and effective for a range of psychiatric diagnoses, including anxiety disorders, bipolar disorder, psychosis, substance abuse, post-traumatic stress disorder, eating disorders, and personality disorders [24, 25, 27, 30, 31]. As a result of its small but promising evidence base, FT has been implemented, in recent years, in several countries (e.g. the USA (Chicago), the Netherlands, Greece, Scandinavia (Norway, Sweden, Finland), and Australia (Victoria)) to support children and families when a parent has mental illness [32].

By contrast, there is a lack of policy guidance and service and public awareness in the RoI on the need to support families where there is parental mental illness [33, 34]. Current mental health service provision is characterised by an individualised, crisis-oriented approach to assessment/treatment; a lack of collaboration between Adult Mental Health Services (AMHS) and Child and Adolescent Mental Health Services (CAMHS); and competency and confidentiality concerns amongst mental health professionals who may feel ill-equipped to undertake family work [9]. In order to address these shortcomings, the national Health Service Executive (HSE) provided funding for the current research programme—called ‘PRIMERA’ (Promoting Research and Innovation in Mental hEalthseRvices for fAmilies and children), the primary aims of which are to (1) identify/develop, implement, and evaluate family-focused interventions for families with parental mental illness and (2) inform a ‘think family’ care delivery agenda within mental health services in Ireland.

In the early stages of the PRIMERA study (2017–2018), the research team conducted a scoping study of service supports for COPMI in the RoI and found that, across adult and child mental health services, family-focused practice (FFP) was either small-scale, ad hoc, or, more typically, non-existent [9]. Barriers included staffing shortages in the mental health services, siloing of adult and child supports, and parental fears of involving child protection services, stigma, and common but inaccurate beliefs that their children do not notice their symptoms. Despite little existing FFP in the RoI, there was considerable enthusiasm to enhance service provision for this population. Following consultation with stakeholders from 2017 to 2019 and a lengthy installation and implementation phase, it was agreed that clinicians across several AMHS and CAMHS sites would deliver Family Talk as part of an RCT (with embedded process and cost evaluations) [9].

The current study offers a significant contribution to the field. Firstly, outcomes from only four RCTs and one quasi-experimental trial of FT have been published to date, with sample sizes of 28, 37, 105, 109, and 37 respectively [23–27]. Therefore, the evidence base for FT is promising but small. Secondly, we will assess family functioning as a primary outcome. So far, only one published study [25] has reported on family functioning despite enhanced family communication and cohesion being key goals of FT [23]. Thirdly, three of the RCTs were evaluated by the programme developer within a controlled ‘efficacy trial’ setting [23–25], whereas the current study will undertake an independent effectiveness trial, using ‘lay’ practitioners with real-world workloads. Lastly, very little qualitative research has been undertaken to investigate the experiences of stakeholders in receiving/providing FT or in conducting costs analyses, the latter of which are often a critical consideration for governments when allocating funding and resources [17]. Indeed, this last criticism applies to the evaluation of family-focused interventions in general, and not only FT.

### The current study: objectives

The current (PRIMERA) study involves an RCT design with embedded process and costs evaluations, in line with the framework of the Medical Research Council for complex interventions [35]. Its principal aims are to assess the clinical and cost-effectiveness and implementation mechanisms of FT for families where a parent has a mental illness, when compared to routinely available supports. Usual services involve the parent with mental illness receiving treatment (e.g. psychotherapy, pharmacotherapy) in AMHS or, in a small number of cases, receiving medication/treatment from their general practitioner (GP). Families in the intervention group will receive FT in addition to usual services. Families allocated to the control group will be put on a waitlist for FT and will be offered the intervention following the 6-month follow-up assessment.

The primary hypotheses are that FT will improve child psychosocial functioning and family functioning. Secondary hypotheses include positive effects on understanding and experience of parental mental health challenges, parental mental health, child and parental resilience, partner wellbeing, and service utilisation. The process evaluation will assess the experiences and views of families/service providers in receiving/delivering FT, including in particular, the barriers and facilitators to implementation. The economic appraisal will assess the cost-effectiveness of FT in improving child and family outcomes compared with usual services.

## Methods

The study protocol has been reported in accordance with the Standard Protocol Items: Recommendations for Clinical Interventional Trials (SPIRIT) guidelines [36] (Additional file 1).

### Trial design

The current study is designed as a multi-centre, randomised, controlled, investigator-blinded superiority trial with two parallel groups and a primary endpoint of child psychosocial functioning and family functioning at 6-month follow-up. Randomisation will be performed as block randomisation with a 2:1 allocation (intervention: control).

### Study settings

Family Talk will be implemented in 15 sites across the RoI, including 10 sites with the statutory HSE adult, child, and primary care mental health services, four sites with the statutory child welfare and protection body (Tusla), and one site with the AMHS in the Saint John of God’s Hospitaller Ministries. Sites are located in both urban and rural areas with catchment populations from a range of socioeconomic backgrounds. A list of study sites can be seen on the PRIMERA website (https://cmhcr.eu/primera-programme/). Across all organisations, FT will be delivered within community outpatient clinics, with a small minority (< 10%) taking place in the home setting.

Most parent participants (< 80%) across sites will be a current patient of AMHS and will be under the clinical responsibility of the consultant psychiatrist / multi-disciplinary team (MDT). In some cases, a family may be involved with CAMHS or with Tusla, but the parent must have a formally diagnosed mental illness and be in receipt of treatment from their GP. Clinical responsibility will be provided by the GP and the service provider of FT. We will recruit only children aged 5 years and over because FT is targeted at children who are able to verbally express their experiences [23].

### Participants and eligibility criteria

Families are eligible for inclusion according to the following criteria:

- Parent(s) aged over 18—and with children aged 5–18 years—attending AMHS and who are under the care of a psychiatrist/MDT due to a formal (or working) diagnosis of mental illness; **OR**
- Parent(s) with a mental illness episode in the last 18 months who had been under the care of a psychiatrist or MDT; **OR**
- Parent(s) currently attending a GP for mental illness.

It should be noted that whilst children over 5 years can receive the FT intervention, data will only be collected from child participants aged 8–18 years, as the selected outcome measures are only suitable for this age group.

### Exclusion criteria for families

Families will be excluded if the parent/family is in a state of crisis/instability such that they cannot engage with the intervention or the research process. This includes:

- Parents/children with active psychosis
- Parents with active substance misuse and unable to engage with FT
- Parents or children who are in hospital
- Parents or children with intellectual disability that affects their capacity to engage with FT or the research process (e.g. completing questionnaires)
- Dispute over child custody
- Urgent need for child protection services.

Where hospitalisation or relapse occur during the delivery of FT, the clinician must make a judgement whether the intervention can be merely postponed or delivered at a later stage when the patient is more stable.

### Eligibility criteria for staff delivering family talk

Clinicians delivering FT must have at least 3 years’ experience in working with adult or child mental health and/or welfare and protection services. All clinicians must also have completed the online training in FT (www.emergingminds.com.au) and receive monthly supervision in FT delivery.

### Intervention

**Fig. 1:**
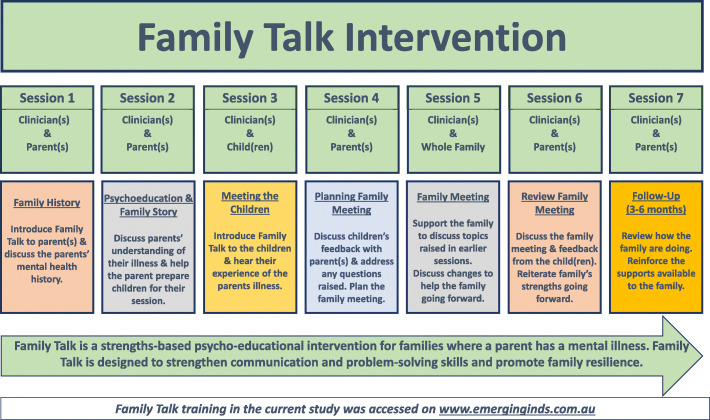
Family Talk sessions

Family Talk is a manualised, strengths-based, 7-session, weekly programme for families where one or both parents have a mental illness. FT uses an individual family format and the trained clinician meets with parents and the children (and extended family members—e.g. grandparent—if requested by the parents). FT is based on psycho-education, narrative, and systemic therapy and is designed to promote family communication and understanding about mental illness and to enhance family resilience and social supports [32]. The first two sessions involve the clinician and parents and includes a discussion of the family’s experience of mental illness and psycho-education, as required. In session 3, the clinician meets with the children alone to conduct an assessment and to identify any questions which the child(ren) may have in relation to their parent’s mental illness. Next, a planning meeting between the clinician and parents is held, after which a whole-family session is organised to support family discussion and provide information on mental disorders as required. The intervention concludes with follow-up meetings (after 1 week and after 3–6 months) to check in and support the family going forward. Each session lasts 60–90 min (Fig. 1).

### Adherence to intervention protocol

Evidence suggests that more positive outcomes of behavioural and psychosocial programmes tend to be associated with higher levels of adherence/fidelity to intervention protocols [37]. The manualisation, and training and supervision of FT, should help ensure consistency and quality of programme delivery. Clinicians must complete weekly session checklists to check that content is covered and must record family attendance and engagement. In addition, as part of the process evaluation, clinicians must complete an online Fidelity Questionnaire for each family that receives FT to capture overall adherence, attendance, and family engagement. The process evaluation will also undertake semi-structured interviews with families and clinicians to assess the degree to which sites implemented FT with fidelity. Clinicians will document if the intervention needed to be modified or discontinued for any reason, including, for example, perceived harm or withdrawal of participant consent.

### Waitlist control group

Families assigned to the control group will receive treatment as usual, i.e. the parent with mental illness will receive medication/psychotherapy from their psychiatrist/MDT/GP. The only difference in service utilisation between the intervention and control group will be that the former will receive Family Talk 6 months earlier. A Services Utilisation Questionnaire will be used to document the types of service supports received by control and intervention group families between T0 and T1 (i.e. baseline to 6-month follow-up).

### Outcomes

**Fig. 2:**
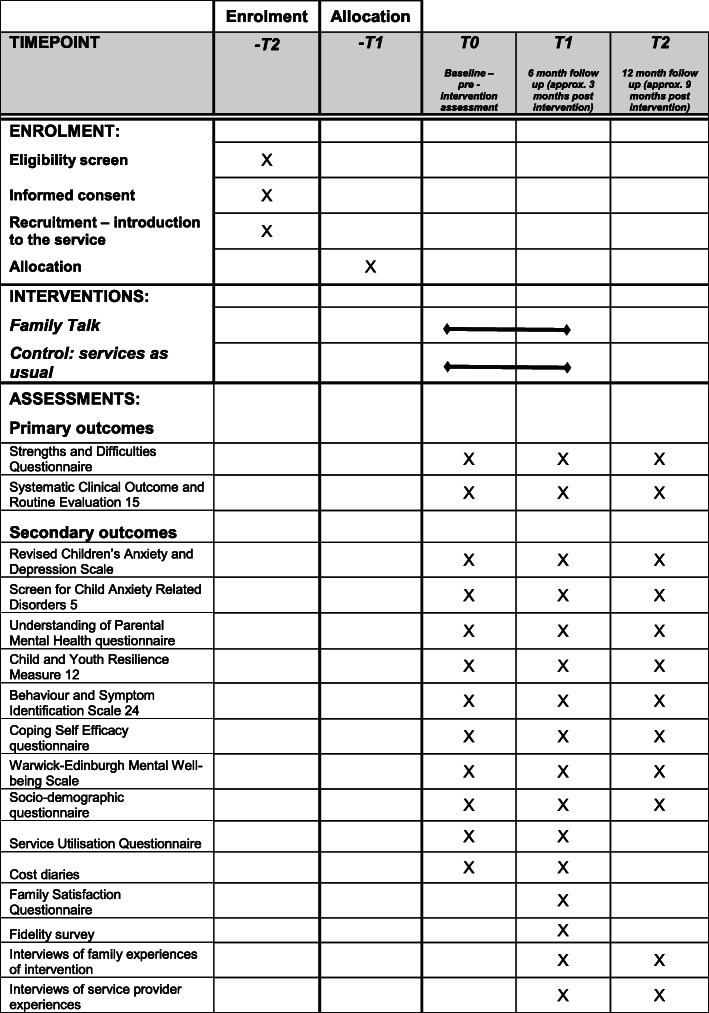
Study flow diagram

This study involves an RCT, and associated costs and process evaluations. The primary and secondary outcomes for the impact evaluation are outlined below, followed by the data collection measures for the costs and process evaluations. All measures for the impact evaluation are based on a continuous scale and will be analysed using covariance adjusted outcomes. All outcome measures for the impact evaluation will be collected at baseline and at 6- and 12-month follow-ups. All children who complete the child-report measures will be over 8 years old. Figure 2 provides an overview of primary and secondary outcomes and data collection points.

### Primary outcomes

There are two primary outcomes: child psychosocial functioning and family functioning.

*Child psychosocial functioning* is measured by the Strengths and Difficulties Questionnaire (SDQ) [38]. The SDQ is a 25-item, widely used, and psychometrically sound questionnaire of child conduct problems, hyperactivity, emotional symptoms, peer problems, and pro-social behaviour for 3–18 year-olds. The SDQ has a parent and child version; in the former, the parent reports on the child’s behaviour whilst in the latter, the child (8–18 years) reports on their own behaviour. Both parents and children will complete the SDQ in the current evaluation.

*Family functioning* is assessed with the Systematic Clinical Outcome and Routine Evaluation (SCORE-15), a 15-item reliable and validated self-report measure of family communication, relationships, and functioning [39]. The SCORE-15 has three dimensions: strengths and adaptability; overwhelmed by difficulty; and disrupted communication. It is validated for use with adults from clinical populations and the child version is suitable for children aged 8–18 years old [40].

### Secondary outcomes

Data will be collected for the following 7 secondary outcome measures:

- *Child depression* will be assessed (via parent and child report) using the 10-item Major Depression subscale from the Revised Children’s Anxiety and Depression Scale (RCADS) [41]. Both the parent and child versions show high internal consistency and convergent validity and have been shown to accurately assess anxiety and depression symptoms in both clinical and school-based youth [42].
- *Child anxiety* will be assessed by parent and child report using the 5-item version of the Screen for Child Anxiety Related Disorders (SCARED-5) [43]. Both the parent and child versions of the SCARED and SCARED-5 demonstrate robust psychometric properties. The SCARED-5 measures generalised anxiety disorder, panic/somatic, separation anxiety, social phobia, and school phobia. A cut-off of 3 can discriminate anxiety from non-anxiety.
- *Understanding and experience of parental mental illness* will be measured using the Understanding of Parental Mental Health questionnaire, via parent and child report. This 20-item questionnaire has been devised specifically by the PRIMERA research team in the absence of any psychometrically robust and validated measures to assess a parent’s understanding and experience of how their mental health affects their children, or to assess a child’s understanding of parental mental illness. A key objective of FT is to improve knowledge and understanding of parental mental health difficulties amongst family members and is therefore an important proximal outcome of the intervention. The questionnaire assesses mental health literacy (e.g. awareness of common myths about mental health), the experience of living with (a parent with) mental illness, and their perceived level of family, social, and service supports.
- *Child resilience* will be assessed with the Child and Youth Resilience Measure 12 (CYRM-12), a brief, validated, 12-item, child-report measure of the child’s individual traits, relationship to caregiver(s), and contextual factors that facilitate a sense of belonging and resilience [44].
- *Parental mental health* will be assessed with the BASIS-24 (Behaviour and Symptom Identification Scale 24), a 24-item, parent-report questionnaire of mental health functioning in clinical populations (aged > 18) across six major areas: depression/functioning, relationships, self-harm, emotional lability, psychosis, and substance abuse [45].
- *Parental coping and resilience* will be measured with the Coping Self-Efficacy questionnaire (CSE), a 26-item self-report measure of parental confidence in performing coping behaviours when faced with life challenges (i.e. use of problem-focused coping, ability to stop unpleasant emotions and thoughts, and receipt of support from friends and family) [46].
- *Partner mental health and wellbeing* will be measured using the Warwick-Edinburgh Mental Well-being Scale (WEMWBS). The WEMWBS is a 14-item, psychometrically robust, self-report measure used to assess mental health and wellbeing in the general population (during the previous 2 weeks). It assesses both hedonic (e.g. life satisfaction) and eudemonic (e.g. psychological functioning and self-realisation) perspectives and also incorporates positive affect (e.g. optimism) and positive functioning (e.g. energy) [47].

### Socio-demographic questionnaire

Demographic and background information on families will be collected at baseline from parents and will include the physical and mental health of family members, perceived social support, and socioeconomic status (assessed using information on parental income, employment, education, and living circumstances). The questionnaire will provide important data for purposes of attrition analysis and for testing the equivalency of the control and intervention groups. A brief follow-up version will be used to assess any changes at 6- and 12-month follow-up (e.g. change of partner, new children)

### Process evaluation

The process evaluation will run in parallel to the RCT and will involve the application of mixed methods to investigate the experiences and contextual factors related to receiving/implementing FT. The analysis will be informed by the MRC guidance for complex interventions [35] and will be located within an implementation science framework (e.g. Fixsen’s implementation model [48]). The quantitative and qualitative elements of the process evaluation are outlined below.

#### Quantitative element: survey and questionnaire

- A *Family Satisfaction Questionnaire* will ask families (parents and children) to assess their experience of FT following the intervention. The parent version of the questionnaire has 18 items and the child version has 12 items. Participants will be asked to evaluate the extent to which FT was a positive experience for families, helped with family functioning and understanding of mental health, and to provide suggestions for improvement.
- An online Qualtrics survey will be administered to clinicians following FT delivery in order to assess implementation fidelity. The survey takes approximately 15 min to complete and assesses clinician adherence to session checklists as well as family attendance and engagement to the intervention.

#### Qualitative element: interviews and focus groups

##### Interviews with parents and partners

Semi-structured interviews will be conducted with a subsample of parents (*n* ≈ 15–20) and partners (*n* ≈ 10) involved in the RCT in order to explore their experience of participating in FT and to learn to what extent it met their needs. A maximum variation sampling strategy will be employed in order to recruit a heterogeneous sample across different site locations and with different demographic characteristics (e.g. age, marital status, socioeconomic status). Interviews will be conducted at 6- and 12-month follow-up. Interviews will take place in the participant’s home/site centre or remotely (phone/video call) and will last approximately 20–40 min. A brief interview will also be carried out with parents who dropped out of FT before completion (*n* ≈ 10) to assess their reasons for attrition.

##### Interviews with children

Semi-structured interviews (15–30 min) will also be conducted with a small sample of children aged 12–18 years (*n* ≈ 10) at 6-month follow-up in order to explore their experience of the FT programme and the extent to which they think it helped them and other family members.

An alternative interview story approach will be used to interview younger children (8–11 years) (*n* ≈ 8–10). The interview story (15–30 min) involves a family imagined by the child in which a parent has mental health difficulties. The child is encouraged to build up a picture of the house, location, and family members who live in the home. Picture cards are used to help the child identify emotions. The child is also asked to imagine that the family attends FT and to relate the family’s experience of the programme (e.g. what helped, what could be improved). The interview story approach has been used successfully in previous research [49] to discuss sensitive issues with younger children (e.g. children who have experienced domestic abuse). The indirect approach allows the child to externalise the issue and talk about it in a more objective fashion, thereby offering emotional protection to the child. Occasionally, children may slip into ‘I’ language during the course of an interview rather than use the name of the character they have chosen. If that happens, the researcher mirrors the language used by the child. Ethical issues in interviewing children are outlined later.

##### Interviews/focus groups with clinicians, managers, and policymakers

One-to-one semi-structured interviews and/or focus groups will be conducted with FT clinicians/service providers across all participating sites (*n* ≈ 20). Individual interviews will last 30–60 min and focus groups will last approximately 60–90 min. This will cover topics such as the experience of implementing and delivering FT within services; key outcomes and enablers and barriers to delivery; rates of recruitment and attrition and organisational support, amongst other issues.

A small number of in person/phone/video call interviews (15–30 min) will also be held with managers/decision makers within sites where possible (e.g. Area Managers, Executive Clinical Directors, consultant psychiatrists) (*n* ≈ 10). The purpose of the interview will be to elicit their experience/views of FT within their service and to assess to what extent the intervention fits with their policy and practice objectives.

### Cost-effectiveness analysis of family talk

A Service Utilisation questionnaire (SUQ) and cost diaries will be used to conduct a cost-effectiveness analysis of Family Talk compared to usual services on our primary outcome measures. The SUQ is an adapted version of the Client Service Receipt Inventory [50] and has been used in previous research [51, 52]. The form will assess the type and number of contact families have had with healthcare, social care, and educational services in the previous 6 months and will be completed at baseline and at 6-month follow-up. The form will be administered by means of a face-to-face interview carried out by researchers with parent participants, along with the other psychometric inventories outlined above.

Cost diaries will be administered to programme facilitators asking them to document the time taken to train in FT, recruit and engage families, and deliver the programme, materials, and costs incurred. Time will be multiplied by salary/wage costs to calculate the approximate cost of delivering FT.

### Participant timeline

The flow of participants from recruitment through to the end of the study is shown in Fig. 2. Parents and children will be assessed at T0 (pre-intervention), at 6-month follow-up (T1; 3 months post intervention), and at 12-month follow-up (T2). Assessment of the control group will continue up to T1, after which they will be offered FT. Assessment of the intervention group alone will continue at 12-month follow-up. Enrolment into the study will be on a staggered basis and will depend on clinicians referring suitable families to the research team.

Data collection and delivery of the intervention were necessarily paused for 4 months (mid-March to mid-July 2020) due to the COVID-19 lockdown restrictions in the RoI. Therefore, assessments affected by the restrictions will be collected 4 months later than originally planned. However, taking the pause into account, we still consider the assessment time points to be 6- and 12-month follow-up. Assessments affected by the COVID-19 suspension will be noted in the analysis.

### Sample size

A power analysis was conducted using the two primary outcome measures—the Strengths and Difficulties Questionnaire (SDQ) and the Systematic Clinical Outcome and Routine Evaluation (SCORE-15)—in order to identify the minimum sample size required to detect an improvement in child and family psychosocial functioning post intervention.

Three of the previous RCTs of FT employed intensive clinician interviews to assess child psychosocial functioning and reported statistically significant results with varying sample sizes (*n* = 28–105) [23–25]. Similar to two previous evaluations of FT [26, 27], this study intends to use the brief SDQ to measure child psychosocial functioning. These studies reported effect sizes of 0.70 and 1, respectively, at post intervention. The Finnish study [26] involved 109 families and the German study [27] recruited 37 families. The power calculation for the current study was conducted for an independent samples *t*-test, as this is expected to be the least powerful test in the overall main analysis. Using G*Power *t*-test calculations for the difference between two independent means (two groups), and assuming *α* = .05, 80% power, two tailed testing, 15% attrition, and 2:1 allocation, the study will need to recruit 38 participants (FT = 25, TAU = 13) to detect a change between 0.7 and 1. Given that the effect sizes in these two studies are large, we intend to recruit additional families to enable us to detect smaller changes, where they exist.

Family functioning has not been typically assessed in previous evaluations of FT despite the fact that enhanced family communication and cohesion are key intervention goals. One previous study has assessed family functioning using intensive clinician interviewing of families [25], although it should be noted that two ongoing evaluations of FT have included it as an outcome, albeit using measures that take considerably longer to administer than the SCORE-15 [28, 29]. The SCORE-15 has been used as a brief measure of family functioning in evaluations of family therapy with populations that share a clinical profile similar to the sample in the current study [53]. An effect size of 0.5 was reported. Similar to above, we conducted a G*Power *t*-test calculation for the difference between two independent means (two groups) for the outcome of family functioning. Assuming *α* = .05, 80% power, two tailed testing, 15% attrition, and 2:1 allocation, the study will need to recruit 144 participants (FT = 96, TAU = 48) to detect a change of 0.5.

These sample sizes should be more than sufficient for mixed model repeated measures (MMRM) across the three time points to have > 80% power and also allow for post hoc *t*-tests both between groups and within groups to have ≥ 80% power.

### Recruitment

The recruitment phase opened in March 2019 and closed on 30th November 2020 to allow sites time to recruit a sufficient sample of suitable families to the research. Unfortunately, the funding timeline means that we cannot further extend the enrolment period in order to account for the 4-month suspension caused by the COVID-19 lockdown restrictions, and as such, recruitment rates will be affected. As indicated above, the early phase of the current study involved a prior installation and implementation period in order to allow sites to train and gain experience in delivering FT [9]. Families (parents and children 5–18 years) will be recruited by clinicians in each site from their existing waiting lists. Recruitment brochures and posters will be used to inform families and clinicians about FT and the PRIMERA research. In addition, each site has nominated a lead contact person who will take responsibility for promoting FT and the PRIMERA research within their area in order to encourage referrals.

Clinicians will meet with families to assess their suitability for FT and the research process using the eligibility criteria outlined above. Once the clinician has secured agreement from the parent that they are interested in participating, they will ask for their written consent (on the family recruitment leaflet) for their contact details to be passed in confidence to the research team. Parents will then be contacted by the research team via telephone to arrange for one of the research team to talk to them in more detail about the study and to arrange to visit them to tell them more about the research and obtain their written informed consent in person. Two children per family—aged 8–18 years—are eligible to participate and their written assent will be obtained once their parent has provided prior consent for their child’s participation. Written informed consent/assent will be obtained at each data collection time point, and also if a parent/child is asked to participate in a qualitative interview. Families will be incentivised to participate with a shopping voucher worth €20 at each data collection visit. Families will be informed within 2 weeks about their allocated group.

Given the traditional segregation of adult and child mental health services in Ireland, it is expected that some clinicians and managers will not see a family-focused service as fitting their role and remit. Therefore, throughout the study, we will spend considerable time on awareness raising activities and liaison with mental health professionals (via meetings, stakeholder events) around the need to provide family-focused mental health services to support this neglected population. We will monitor the referral rates across all sites and will problem-solve with clinicians on strategies to increase recruitment if and when such issues emerge. Collectively, the research team have considerable experience of working with vulnerable and difficult-to-engage populations, and their expertise, in conjunction with the advice and support of collaborating sites, will be important in managing the recruitment process.

### Sequence generation and allocation concealment

Once the family is enrolled and baseline evaluations are completed, the family will be randomised to either the intervention arm or a waitlist treatment as usual group by an external consultant (NW) who will be blind to evaluation outcomes and will conceal the allocation sequence until the intervention is assigned. Randomisation will not be influenced by the research team or practitioners involved in FT delivery. Randomisation will be stratified by site with families allocated to either the intervention or control group on a 2:1 basis using the SNOSE (sequentially numbered opaque sealed envelopes) method [54]. The use of block randomisation means that the intervention can be delivered in a staggered manner, with some participants beginning the intervention whilst further recruitment continues. The external consultant will inform the research team of any issues arising relating to randomisation (e.g. failure to contact clinician to inform of randomisation outcome). Practitioners who deliver the intervention are not involved in delivering services to the control group (and vice versa) in order to ensure that there is no contamination between the intervention and control groups.

### Blinding

Due to the nature of the intervention, neither participants nor practitioners can be blinded to allocation. Outcome assessors will be blinded to treatment allocation, as well as those conducting the statistical analyses. Randomisation will be conducted by an external consultant who is unconnected to the recruitment, data collection, or analysis process. Participants (families), and practitioners will be requested not to disclose their group allocation to the research team. Any evidence of unblinding will be taken into account at the analysis stage.

### Data collection

#### Families

Participants will be invited to complete outcome measures at baseline (T0) and at 6- and 12-month follow-up periods (T1 and T2) (Fig. 2). Each questionnaire will be coded with the participant’s ID number, date of completion, version (baseline, T1, T2), and the researcher’s name. Qualitative data (i.e. interviews) will be collected from families following the 6-month assessment within the RCT. Copies of the impact evaluation and costs measures, and interview schedules for the process evaluation, can be obtained from the PRIMERA programme coordinator (https://cmhcr.eu/primera-programme/).

For all aspects of the evaluation (RCT, costs and process evaluations), data will be collected by a researcher who will meet with the participant in the family home, or, if preferred, in a local family/health care centre. Data will also be collected from parents by video call where it is not safe, due to COVID-19, to meet in person. Parents and children (8–18 years) will provide written informed consent/assent prior to data collection and will be assured of ethical protections (e.g. confidentiality) and risks and benefits from participation. Parents will complete a paper version of outcome measures and the assessment will take 45–75 min (with a break if required). Whilst electronic devices may sometimes improve data accuracy and the timeliness of data collection and management [55], the experience of the research team and site staff is that vulnerable populations may be alienated by their use and are more comfortable with paper-based data collection methods. Based on feedback from the mental health service professionals with whom we are working, children will be offered an option of completing a paper version of the survey in the presence of the researcher or via the secure online Qualtrics software package [56]. Both options will take 10–15 min. The online survey will include voice-recorded files in order to assist comprehension. The online survey will be accessed via the project webpage in compliance with *General Data Protection Regulation*s (GDPR). No personal identifiers of children will be collected using Qualtrics and Qualtrics does not share data with third parties [56].

For the qualitative interviews, parents and children will provide consent/assent for their interview to be digitally recorded. Anonymity and other ethical protections will be assured (Information sheets and consent/assent forms are available upon request).

In order to enhance the quality of the data, all fieldworkers will receive standardised training in how to administer the measures with families. In addition, selected fieldworkers will be experienced in collecting data from vulnerable populations. Missing, incomplete, or inaccurate data will be assessed within a week of data collection by the data-input manager, who will liaise with the relevant fieldworkers for information. Families will be re-contacted if necessary for any missing data.

Participant retention will be promoted using the following strategies: scheduling appointments by telephone and reminding participants by text; minimising participant burden during visits; financial reimbursement at each data collection point; and liaising with study sites to locate hard-to-reach parents. Every effort will be made by both the research team and study sites to engage families for the entire study period. Where a participant withdraws consent for one follow-up assessment, they will be asked if they would consent to participate at another time point, or for another element of the study (e.g. the process or costs evaluation). All participants will be included in an intention-to-treat analysis, regardless of adherence or non-retention. Reasons for missing data—both non-adherence (e.g. family crisis, perceived harm or non-efficacy) and non-retention (e.g. consent withdrawn; lost to follow-up)—will be sought from parents and practitioners and will be recorded and interpreted during analysis.

#### Data collection—service providers

Semi-structured interviews (30–60 min), focus groups (60–90 min), and video/phone calls (15–30 min) will be audio recorded with participants’ consent and transcribed verbatim and in full. A secure online Qualtrics survey (lasting 15 min) will be administered on a once-off basis only, to clinicians following FT delivery in order to assess implementation fidelity, participant attendance, and engagement.

#### Data management

Quantitative data will be entered into a database (IBM SPSS Statistics version 26) by a data manager who will conduct regular verification checks and quality audits to ensure accuracy of entry and coding. This process will be overseen by the Programme Coordinator. The data manager will also liaise with fieldworkers to clarify the accuracy of entered data. Qualitative data (i.e. interview and focus-group data) will be transcribed verbatim and stored on a secure, central network, which is encrypted and password-protected. Data will be coded and analysed using the qualitative analysis software package MAXQDA [57].

The study will adhere closely to the GDPR and data protection guidelines on research in the health sector by the Data Commissioner of Ireland [58]. All research staff have received GDPR training. All forms will be anonymised through the allocation of a unique identification number and will be stored under lock and key throughout, and following completion of, the study. All computers will be password-protected and the transfer of any information from laptops to the secure central network will be carried out with extreme caution. Encryption software will also be used to encrypt sensitive data which are stored electronically. Encrypted data will be accessible using a key which will only be known to the research team members. A separate (password-protected) database of names and contact details will be stored away from the other data and there will be no means of linking the two.

Where participants provide consent/assent, an anonymised version of their data will be placed in the Irish Qualitative Data Archive (IQDA) and the Irish Social Sciences Data Archive (ISSDA) so that it may be used by future researchers if so required. Future researchers will require ethical approval for their study to proceed. In all other instances, data will be destroyed 10 years after the completion of the study; manual data will be shredded confidentially and electronic data will be reformatted or overwritten.

#### Statistical methods

Data presentation and results will be carried out according to Consolidated Standards of Reporting Trials (CONSORT) guidelines for RCTs. Descriptive statistics (means, standard deviations, frequencies) will be used to describe the pre-treatment characteristics of participants and for primary and secondary outcome measures at each time point. Mixed model repeated measures (MMRM) will be used to investigate the effects of the intervention at two between (intervention and control) and three within (pre-intervention, 6- and 12-month follow-ups) levels for all primary and secondary outcome measures (all continuous outcome data). Initial MMRM analysis will control for baseline psychopathology as a fixed covariate. Modelling for the primary outcomes will be conducted using an unstructured repeated measures covariance matrix and all other variables as fixed effects. Mean difference effect sizes, 95% CIs, and *p* values will be reported for continuous outcomes. Where parametric test assumptions fail significantly, then non-parametric tests will be used.

MMRM was chosen as the primary method for analysis as it can reduce several analytic problems that may arise within the current study. Firstly, it allows for different numbers of measurements per participant, thereby tolerating a level of missing data, which is particularly problematic with RCTs with vulnerable populations, as follow-up data are often collected many months after treatment has ended and participants may be difficult to contact [59]. This enables us to use all of the data collected as opposed to deleting cases or imputing missing values. Secondly, it has the advantage of modelling change within individuals as well as across groups. Singer and Willet [60] identify this as the best approach for longitudinal data that has three or more time points.

The analysis will follow an intention-to-treat (ITT) principle where all randomised participants, including those who stop receiving the intervention, will be analysed ‘as randomised’. MMRM analysis is a maximum likelihood statistical modelling technique whereby mean estimates and the repeated measures covariance structure for the observed data are based on a statistical model and possible values are generated for the missing data [61]. Attrition will also be analysed to assess the differences between those who ‘dropped out’ and those who stayed, and to assess if there are predictors at baseline to indicate differences. Qualitative data will also inform the identification of predictor variables for attrition. MMRM will be the primary analytic method, although *t*-tests will be used to provide detail on any significant differences found from the MMRM.

Regression techniques will be used to explore whether intervention effects differ for certain participant groups such as age of children, severity of parental mental illness, other parent also has a mental illness, and socioeconomic status. Analysis will be conducted using SPSS software (IBM SPSS Statistics version 26).

#### Process evaluation analysis

The qualitative data (interviews, focus groups, meeting minutes) will be coded and organised using MAXQDA and analysed using constructivist grounded theory [62]. The analysis will be informed by the MRC framework for process evaluations and located within an implementation science framework [35, 48]. One researcher will code all the data, with inter-reliability of codes checked by another researcher on 25% of transcripts selected at random. Any differences encountered by the researchers will be discussed and resolved between them.

We will use descriptive statistics to analyse measures of fidelity (therapist adherence, family attendance), and the Family Satisfaction Questionnaire. Frequencies, medians, interquartile ranges, means, and standard deviations will be reported as appropriate.

#### Economic evaluation

The economic evaluation will compare the cost-effectiveness of receiving FT versus a services as usual control group on our primary outcomes at 6-month follow-up. The cost-effectiveness analysis CEA will be undertaken in three key steps:

(i) Identify the cost of delivering FT, obtained through cost diaries completed by service providers
(ii) Compare service utilisation for the intervention and control groups conditions (completed by parents on the Services Utilisation Questionnaire at baseline and 6-month follow-up); and
(iii) Calculate an incremental cost-effectiveness ratio (ICER) to give the cost of obtaining a one unit decrease on the clinical outcome measures employed in the RCT (i.e. SDQ, SCORE-15) when comparing FT to usual services at 6-month follow-up.

The ICER will use a 1000 replication bootstrap to provide a 95% confidence interval accompanied by appropriate sensitivity analyses (e.g. excluding non-recurrent costs of training). The ICER accommodates sampling (or stochastic) uncertainty and varying levels of willingness to pay for reductions in the primary outcomes of interest. The CEA will adopt a multiagency, public sector, analytic perspective and will use official sources (e.g. government pay scales, the Casemix/HIPE unit of the Health Service Executive) to provide estimates of unit costs separate from frequencies of resource utilisation.

#### Monitoring

A data monitoring committee is not feasible for this trial due to its relatively short duration and size. Only the research team will have access to the data. The data-input manager will ensure that the quality of data is maintained throughout the trial. Only the data-input manager and the team statistician will have access to the outcome data and they will be blind to study groups. The fieldwork coordinator will inform the research team of any issues arising with participant recruitment and data collection.

Due to the relatively short duration of this trial, we will not conduct an interim analysis as sufficient data will not be available at that point. The trial will only be stopped by the principal investigator (in conjunction with the PRIMERA funders) if it becomes clear that harm is being caused by the intervention or recruitment rates are unacceptably low.

The research team will report quarterly to the study’s Steering Group on trial conduct and progress. The Steering Group comprises high-level management from the HSE and Tusla, service users, and representatives from mental health advocacy groups and the academic sector.

#### Harms

Based on previous evaluations of FT, we do not anticipate any harm or adverse effects from participation in the intervention. Families are informed that they are free to withdraw at any stage if they feel that FT is not beneficial for them. Service providers will stop the intervention if they (in conjunction with the family) decide that it is causing any harm. If a parent suffers a relapse in mental health symptoms, or there is a crisis in the family during the delivery of FT, it is recommended that programme delivery is postponed until the family is in a position to re-engage. In all sites, service providers will have clinical responsibility for patients and families involved in the intervention.

We do not anticipate harm arising from the research process; based on our previous experience, it is envisaged that most participants will find the interview sessions helpful. Nevertheless, we indicate in our information sheets that parents with mental health issues may be potentially vulnerable in terms of the sensitivity of the issues discussed in data collection (e.g. history of mental health challenges) and may possibly feel emotionally distressed as a result. In addition, there is a potential risk that parent welfare concerns may arise during the discussions; for instance, it may emerge during the interview process that a parent may need to link into services with regard to their mental illness (e.g. as indicated by their score on the BASIS-24). If a parent welfare concern arises during the interview process, that person will be directed by the researcher to the FT service provider, who will refer the person to their GP, mental health professional, or consultant psychiatrist, as appropriate.

In addition, the study will include dependent children who have a parent with mental health issues. During the research process, we will be highly attentive to child welfare and protection concerns and will follow Children First guidance [63]. All research staff have received training in Children First guidelines. As in the case of parents, we will also be sensitive to any signs of child discomfort and distress during interviews. The information sheet for families, and every page of our online survey for children, provides advice and contact supports in the unlikely event that children may feel distressed at any point during, or following, a survey or face-to-face interview.

Our ongoing contact with parents and service providers, as well as the qualitative interviews, will alert the research team to any unanticipated harms from the intervention. In addition, unexpected negative effects (e.g. if child and parent symptoms increase following FT) may be detected on our outcome measures which will be carefully reviewed after every assessment.

#### Auditing

Auditing will not be necessary in this study due to its short duration.

#### Ethics

The trial has received approval from four ethics committees: the Social Research Ethics Committee in Maynooth University (Reference number SRESC-2018-100, the HSE Research Ethics Committee, Tusla Ethics Review Committee, and the Saint John of God’s Research Ethics Committee. Any amendment to the protocol that affects trial implementation and outcomes will require formal approval by each research committee. The trial registry (e.g. ISRCTN) will be notified of any substantive modifications to the protocol.

##### Consent and assent

Clinicians in each site will verbally inform the service user parent about FT and the PRIMERA research. Once the clinician has secured agreement from the parent that they are interested in participating, they will ask for the parent’s written consent (on the family recruitment leaflet) for their contact details to be passed in confidence to the research team. Parents will then be contacted by the fieldwork coordinator via telephone to discuss the research in more detail, and to arrange for a member of the research team (fieldworker) to visit the family and obtain their written informed consent to participate. Children aged 8–18 years are eligible to participate in the research once their parent has provided prior written informed consent. Parents will be shown a copy of the child’s survey and be given an age-appropriate information sheet to explain to their child. A researcher will also explain the research to the child. Children who decide to participate will then provide their informed written assent. Written informed consent/assent will be obtained at each data collection time point (e.g. baseline, 6- and 12-month follow-ups, and also if a parent/child is asked to participate in a qualitative interview).

Service providers (clinicians, management) within each site will also be invited to participate in semi-structured interviews/focus groups and will be asked to provide written informed consent. They will be asked to provide their informed consent too before completing the brief online survey on implementation fidelity.

##### Confidentiality

All participants in the study (parents, children and service providers) will be assured of confidentiality and anonymity of their data throughout the research process, including their data protection rights of secure storage, processing, and deletion of their data. Participants will be informed of limits to confidentiality (where there is a risk to participant or child welfare) in the information sheet and consent form. No identifiable data will be collected using the Qualtrics online platform, and Qualtrics does not share data with third parties [57]. In addition, participants will be informed that they may withdraw from the study and/or withdraw/amend parts of their data if they so wish. They will also be informed that the results will be presented in anonymised form at conferences and published in reports and scientific journals. With their consent, an anonymised version of their data will be archived in IQDA and ISSDA**.** Researchers who wish to access these data will require separate ethical approval from an approved institution.

Study data files will be encrypted and uploaded onto a central, password-protected, secure site at X University that is accessible only to the research team. Data and identifier information will be held separately. The coding key and unarchived data (manual and electronic) will be destroyed by the PI 10 years following completion of the study.

#### Post-trial care

Families recruited to the trial will be under the clinical responsibility of their consultant psychiatrist/MDT/GP and will continue to be under their care following receipt of the Family Talk intervention.

#### Dissemination

Trial results will be disseminated to participants, funders, collaborating organisations and to a wide range of relevant government departments and other interested organisations (e.g. community- and voluntary-based services). We will also communicate the findings on traditional and social media. To date, we have increased awareness of the research amongst a large number of mental health and child and family services by, for example, hosting several stakeholder events. The study findings will be presented at appropriate academic conferences—both national and international (Covid permitting)—and published in peer-reviewed journals and on relevant websites (e.g. the HSE website, PRIMERA webpage). A number of academic papers are anticipated including (1) a paper on 6- and 12-month outcomes from the study and (2) various papers on the experiences of stakeholders in receiving/implementing the intervention. The results will be submitted for publication regardless of the magnitude or direction of the effect. A summary report of the findings with attendant recommendations for policy and practice will be prepared by the PRIMERA research team for presentation to our funder, the HSE. Anonymised participant-level data, along with statistical codes, will be made publicly available in the ISSDA and IQDA, as required by registration with the ISRCTN. This will be available within 12 months of the trial end date.

## Discussion

Given the prevalence and personal, social, and economic burden of intergenerational mental illness, it is essential that effective evidence-based interventions are identified and upscaled in routine mental health settings [3, 17]. As outlined earlier, the current study will provide an important contribution to the international evidence base for FT in a number of ways, including its RCT design, size, and scope, and the addition of both a process evaluation and economic appraisal. The current study is also one of the few RCTs of FT worldwide and the first of its kind in Ireland where it is a key component of the first systematic national drive to develop and implement FFP for COPMI in an Irish context.

### Strengths

A key strength of the current study is its use of a multi-centre effectiveness trial, involving almost 70 clinicians (e.g. psychologists, social workers, family therapists) within routine service settings and with real-world workloads. In addition, unlike three of the four previous RCTs of FT, the current study is an independent evaluation of FT as the programme developer is not involved in implementation or analysis. Positive outcomes from the trial would support the scaling up of FT across mental health services in the RoI and elsewhere, but even in the absence of such outcomes (or if the findings were to be mixed), the research in and of itself has been, and will continue to be, a key driver in encouraging services in Ireland to incorporate FFPs into their routine service delivery.

Process and economic analyses are not commonly incorporated into evaluations of interventions in this field [17]. The findings from the current process evaluation will provide important insights into the barriers and facilitators of successful service implementation, as well as the mechanisms underpinning service delivery and positive or negative outcomes for participating families. This will be important in terms of generating key lessons for service implementation in other jurisdictions. Additional data on costs will provide an indication of the overall cost-effectiveness, or value for money, of the intervention, an important consideration in upscaling effective interventions and especially within the context of already limited resources within the mental health services in Ireland.

Another strength of the study is the involvement of multiple stakeholder groups as participant informants, including parents, partners, children, and service providers. To date, evaluations of FT (and other similar interventions) have only included parents with mental illness as informants. The inclusion of child informants is important as studies indicate that COPMI may experience considerable confusion, anxiety, and stress which they often conceal from their parents in order not to overload them with their concerns [14]. Moreover, parents often do not openly discuss their mental illness with their children, in the mistaken belief that children are unaware of their problems, or that such discussion would overburden their children [15, 16]. The inclusion of partners is also important as previous studies suggest that they often feel unsupported by mental health services with regard to the care which they provide and would like services to consult and involve them more in the treatment process [64]. Indeed, other literature indicates that persons with mental illness want their partners to be involved in their treatment [65], although this should be an individual choice rather than mandated [66]. Therefore, adding the voices of children, partners, and service providers to those of parents with mental illness, is vital in informing the effective delivery of family-focused mental health practice in mental health settings.

### Limitations

Based on experiences in other countries in implementing FFP, we expect some difficulties in recruiting families to the trial [67]. FFP for this population has traditionally not been practised within mental health settings within the RoI [9]. Whilst participating sites have been given a considerable amount of time to instal and implement FT prior to recruiting participants for the RCT, systemic barriers still exist, including, for example, practitioner workload and turnover, and a persistent perception amongst some AMHS and CAMHS personnel that FFP is a ‘luxury’ preventive issue and that family support is not their priority or their remit. In addition, we anticipate that some parents with mental illness may not be comfortable in coming forward for help, given the double stigma of having a mental illness and revealing struggles in parenting. As indicated earlier, we have already invested considerable time and effort in raising awareness amongst practitioners and management of the need to support this population and to promote FT in their communities. We have also promoted FFP in local and national media through various fora including print and social media, radio, and television. In order to promote family engagement, clinicians are urged, where possible, to offer families a choice in delivering FT either within the local community clinic or family home setting.

The current study is also limited in that we can only conduct a 6- and 12-month follow-up within the allotted funding timeframe. Evidence from previous evaluations suggests that benefits tend to accumulate and improve in the longer term; for instance, Beardslee and colleagues found that child and parent mental health outcomes improved at 4.5 year follow-up compared with the 1.5 year follow-up [25]. However, previous evaluations have reported benefits at post intervention and at 18-month follow-up [26, 27] so it is hoped that differences (if they exist) can be detected at 6- and 12-month follow-up within the current study, and indeed, these time points are commonly used in the evaluation of psychosocial programmes [21].

An unforeseen challenge, in the context of the current study, is the impact of COVID-19. The study has had to be paused for 4 months (March–July 2020) due to the COVID-19 lockdown restrictions in the RoI. This will have a negative impact on recruitment rates and potentially on retention, as several families have contracted COVID-19 and have had to withdraw from the study. In addition, one third of the families recruited, to date, reported that their mental health had worsened as a result of the pandemic, although by the same token, most families were coping well. Furthermore, practitioners are experiencing challenges in resuming clinical practice in terms of their capacity to deliver FT safely including, for example, a lack of suitable rooms in site centres and difficulties in achieving 2-m physical distancing in the family home. Moreover, most MDTs have suffered staff shortages as a result of the ongoing lack of formal and informal childcare supports in the RoI due to COVID-19. The research team are working closely with practitioners and managers to help support them in resuming delivery of FT. Data collection from July 2020 will be undertaken in line with COVID-19 guidelines [68, 69].

In conclusion, the current study will provide an important contribution to the international evidence base in terms of conducting a rigorous effectiveness trial of FT and in producing high-quality data on costs and key implementation factors. Rigorous trials are essential in providing a sound evidence base to inform policy and practice across the world. Given the prevalence and burden of parental mental illness (including the high risk of intergenerational transmission), it is imperative that we identify effective and cost-effective interventions that are capable of being implemented within routine service settings. The current study will also offer a unique perspective on whether it is possible to produce a paradigm change within mental health services and achieve positive outcomes for families within a time-limited national research programme to develop and promote family-focused practice for families where a parent has mental illness.

## Trial status

The recruitment phase of the study started in March 2019. Recruitment finished on 30th November 2020. It is anticipated that the study will be completed by the end of 2021. This is protocol version 4, 06.08.2020, recruitment timeline was amended twice to take account of Covid-19 restrictions.

## Supplementary Information

**Additional file 1.** SPIRIT 2013 Checklist: Recommended items to address in a clinical trial protocol and related documents*.

## Data Availability

The PRIMERA research team and the funder, HSE, will be given access to the cleaned dataset at the end of the study. Where participants provide consent, anonymised data will be made available through the ISSDA and the IQDA as required by registration with the ISRCTN.

## Abbreviations

AMHS: Adult Mental Health Services
BASIS-24: Behaviour and Symptom Identification Scale 24
CAMHS: Child and Adolescent Mental Health Services
CBA: Cost-benefit analysis
CEA: Cost-effectiveness analysis
CI: Confidence interval
CONSORT: Consolidated Standards of Reporting Trials
COPMI: Children of parents with mental illness
CSE: Coping Self-Efficacy questionnaire
CYRM-12: Child and Youth Resilience Measure 12
FFP: Family-focused practice
FT: Family Talk
GDPR: *General Data Protection Regulation*s
GP: General practitioner
HIPE: Hospital In-Patient Enquiry
HSE: Health Services Executive
ICER: Incremental cost-effectiveness ratio
ISRCTN: International Standard Randomised Controlled Trial Number
IQDA: Irish Qualitative Data Archive
ISSDA: Irish Social Sciences Data Archive
ITT: Intention-to-treat
MDT: Multi-disciplinary team
MMRM: Mixed model repeated measures
MRC: Medical Research Council
PI: Principle investigator
RCT: Randomised controlled trial
PRIMERA: Promoting Research and Innovation in Mental hEalth seRvices for fAmilies and children
RCADS: Revised Children’s Anxiety and Depression Scale
RoI: Republic of Ireland
SCARED-5: Screen for Child Anxiety Related Disorders 5
SCORE-15: Systematic Clinical Outcome and Routine Evaluation 15
SDQ: Strengths and Difficulties Questionnaire
SES: Socioeconomic status
SNOSE: Sequentially Numbered Opaque Sealed Envelopes
SPSS: Statistical Package for the Social Sciences
SUQ: Services Utilisation Questionnaire
WEMWBS: Warwick-Edinburgh Mental Well-being Scale

## References

[1] Rasic D, Hajek T, Alda M, Uher R (2014). Risk of mental illness in offspring of parents with schizophrenia, bipolar disorder, and major depressive disorder: a meta-analysis of family high-risk studies. Schizophr Bull.

[2] Maybery D, Reupert A, Patrick K (2009). Prevalence of children whose parents have a mental illness. Psychiatr Bull.

[3] Hosman CMM, van Doesum KMT, van Santvoort F (2009). Prevention of emotional problems and psychiatric risks in children of parents with a mental illness in the Netherlands: I. The scientific basis to a comprehensive approach. Aust e-J Adv Ment Health.

[4] OECD/EU (2018). Health at a glance: Europe 2018: State of Health in the EU cycle.

[5] Central Statistics Office (2016). Census of population 2016 - profile 4 households and families.

[6] Nicholson J, Levin B, Becker M (2010). Parenting and recovery for mothers with mental disorders. A public health perspective of women’s mental health.

[7] Nicholson J, Biebel K, Williams VF, Kkatz-Leavy J. The prevalence of parenthood in adults with mental illness: implications for state and federal policy, programs, and providers. Psychi Pub Pres. 2002;153 http://escholarship.umassmed.edu/psych_pp/153. Accessed 25 July 2020

[8] Grant A, Reupert A (2016). The impact of organizational factors and government policy on psychiatric nurses’ family-focused practice with parents who have mental illness, their dependent children, and families in Ireland. J Fam Nurs.

[9] Mulligan C, Furlong M, McGilloway S (2020). Promoting and implementing family-focused interventions for families with parental mental illness: scoping and installation. Adv Ment Health.

[10] Weissman MM, Wickramaratne P, Nomura Y, Warner V, Pilowsky D, Verdeli H (2006). Offspring of depressed parents: 20 years later. Am J Psychiatry.

[11] England MJ, Sim LJ (2009). Depression in parents, parenting, and children: opportunities to improve identification, treatment, and prevention.

[12] Keshavan M, Montrose DM, Rajarethinam R, Diwadkar V, Prasad K, Sweeney JA (2008). Psychopathology among offspring of parents with schizophrenia: relationship to premorbid impairments. Schizophr Res.

[13] Lizardi H, Klein DN, Shankman SA (2004). (2004). Psychopathology in the adolescent and young adult offspring of parents with dysthymic disorder and major depressive disorder. J Nerv Ment Dis.

[14] Murphy G, Peters K, Jackson D, Wilkes L (2011). A qualitative meta-synthesis of adult children of parents with a mental illness. J Clin Nurs.

[15] Mordoch E, Hall WA (2008). Children’s perceptions of living with a parent with a mental illness: finding the rhythm and maintaining the frame. Qual Health Res.

[16] Somers V (2007). Schizophrenia: the impact of parental illness on children. Br J Soc Work.

[17] Bee P, Bower P, Byford S (2014). The clinical effectiveness, cost-effectiveness and acceptability of community-based interventions aimed at improving or maintaining quality of life in children of parents with serious mental illness: a systematic review. Health Technol Assess.

[18] Caitlin F, James EL, Anderson K, Lloyd D, Judd F (2006). Intervention programs for children of parents with a mental illness: a critical review. Int J Ment Health Promot.

[19] Reupert A, Cuff R, Drost L, Foster K, van Doesum KTM, van Santvoort F (2012). Intervention programs for children whose parents have a mental illness: a review. MJA Open.

[20] Schrank B, Moran K, Borghi C, Priebe C (2015). How to support patients with severe mental illness in their parenting role with children aged over 1 year? A systematic review of interventions. Soc Psychiatry Psychiatr Epidemiol.

[21] Siegenthaler E, Munder T, Egger M (2012). Effects of preventive interventions in mentally ill parents on the mental health of the mental health of offspring: systematic review and meta-analysis. J Am Acad Child Adolesc Psychiatry.

[22] Huntsman L (2008). Parents with mental health issues: consequences for children and effectiveness of interventions designed to assist children and their families.

[23] Beardslee WR, Wright E, Rothberg PC, Salt P, Versage E (1996). Response of families to two preventive intervention strategies: long-term differences in behavior and attitude change. J Am Acad Child Adolesc Psychiatry.

[24] Beardslee WR, Salt P, Versage EM, Gladstone TRG, Wright EJ, Rothberg PC (1997). Sustained change in parents receiving preventive interventions for families with depression. Am J Psychiatry.

[25] Beardslee WR, Wright EJ, Gladstone TR, Forbes P (2007). Longterm effects from a randomized trial of two public health preventive interventions for parental depression. J Fam Psychol.

[26] Solantaus T, Paavvonen EJ, Toikka S, Punamaki RL (2010). Preventive interventions in families with parental depression. Eur Child Adolesc Psychiatry.

[27] Christiansen H, Anding J, Schrott B, Röhrle B (2015). Children of mentally ill parents—a pilot study of a group intervention program. Front Psychol.

[28] de Angel V, Prieto F, Gladstone TRG (2016). The feasibility and acceptability of a preventive intervention programme for children with depressed parents: study protocol for a randomised controlled trial. Trials.

[29] Giannakopoulos G, Tzavara C, Kolaitis G. Preventing psychosocial problems and promoting health-related quality of life in children and adolescents struggling with parental depression. Open J Depress. 2015; 10.4236/ojd.2015.42003.

[30] Pihkala H, Cederström A, Sandlund M (2010). Beardslee’s preventive family intervention for children of mentally ill parents: a Swedish national survey. Int J Ment Health Promot.

[31] Pihkala H, Dimova-Bränström N, Sandlund M (2017). Talking about parental substance abuse with children: eight families’ experiences of Beardslee’s family intervention. Nordic J Psychiatry.

[32] Beardslee WR, Solantaus TS, Morgan BS, Gladstone TR, Kowalenko NM (2013). Preventive interventions for children of parents with depression: international perspectives. Med J Aust.

[33] Grant A, Goodyear M, Maybery D, Reupert A (2016). Differences between Irish and Australian psychiatric nurses’ family-focused practice in adult mental health services. Arch Psychiatr Nurs.

[34] Falkov A, Goodyear M, Hosman CM, Biebel K, Skogøy BE, Kowalenko N (2016). A systems approach to enhance global efforts to implement family-focused mental health interventions. Child Youth Serv.

[35] Craig P, Dieppe P, Macintyre S, Michie S, Nazareth I, Petticrew M (2008). Developing and evaluating complex interventions: Medical Research Council.

[36] Chan A-W, Tetzlaff JM, Gøtzsche PC, Altman DG, Mann H, Berlin J (2013). SPIRIT 2013 explanation and elaboration: guidance for protocols of clinical trials. BMJ.

[37] Mihalic S, Irwin K (2003). Blueprints for violence prevention: from research to real-world settings – factors influencing the successful replication of model programs. Youth Violence Juvenile Justice.

[38] Goodman R (1997). The strengths and difficulties questionnaire: a research note. J Child Psychol Psychiatry.

[39] Stratton P, Lask J, Bland J, Nowotny E, Evans C, Singh R, Janes E, Peppiatt A (2014). Validation of the SCORE-15 index of family functioning and change in detecting therapeutic improvement early in therapy. J Fam Ther.

[40] Jewell T, Carr A, Stratton P, Lask J, Eisler I (2013). Development of a children’s version of the SCORE index of family function and change. Fam Process.

[41] Chorpita BF, Yim LM, Moffitt CE, Umemoto LA, Francis SE (2000). Assessment of symptoms of DSM-IV anxiety and depression in children: a revised child anxiety and depression scale. Behav Res Ther.

[42] Ebesutani C, Reise S, Chorpita BF, Ale C, Regan J, Young J (2012). The revised child anxiety and depression scale - short version: scale reduction via exploratory bifactor modeling of the broad anxiety factor. Psychol Assess.

[43] Birmaher B, Brent DA, Chiappetta L, Bridge J, Monga S, Baugher M (1999). Psychometric properties of the screen for child anxiety related emotional disorders (SCARED): a replication study. J Am Acad Child Adolesc Psychiatry.

[44] Liebenberg L, Ungar M, Van de Vijver F (2012). Validation of the child and youth resilience measure-28 (CYRM-28) among Canadian youth. Res Soc Work Pract.

[45] Eisen SV, Gerena M, Ranganathan G (2006). Reliability and validity of the BASIS-24 mental health survey for whites, African-Americans, and Latinos. J Behav Health Serv Res.

[46] Chesney MA, Neilands TB, Chambers DB, Taylor JM, Folkman S (2006). A validity and reliability study of the coping self-efficacy scale. Br J Health Psychol.

[47] Tennant R, Hiller L, Fishwick R, Platt S, Joseph S, Weich S, Parkinson J, Secker J, Stewart-Brown S (2007). The Warwick-Edinburgh mental well-being scale (WEMWBS): development and UK validation. Health Qual Life Outcomes.

[48] Fixsen DL, Blase KA, Naoom SF (2009). Core implementation components. Res Soc Work Pract.

[49] Furlong M, Leckey Y, O’Connor S, McMahon K. Evaluation of the TLC Kidz programme for children and mothers recovering from domestic abuse. Barnardos. .

[50] Beecham J, Knapp M, Thornicroft G, Brewin C, Wing J (1992). Costing psychiatric services. Measuring mental health.

[51] McGilloway S, Ni Mhaille G, Bywater T, Furlong M, Leckey Y, Kelly P, Comiskey C, Donnelly M (2012). A parenting intervention for childhood behavioural problems: a randomised controlled trial in disadvantaged community-based settings. J Consult Clin Psychol.

[52] Hickey G, McGilloway S, Furlong M, Leckey Y, Bywater T, Donnelly M. Understanding the implementation and effectiveness of a group-based early parenting intervention: a process evaluation protocol. BMC Health Serv Res. 2016; 10.1186/s12913-016-1737-3. 10.1186/s12913-016-1737-3 PMC5025622 27633777

[53] Hamilton E, Carr A, Cahill P, Cassells C, Hartnett D (2015). Psychometric properties and responsiveness to change of 15 and 28 item versions of the SCORE: a family assessment questionnaire. Fam Process.

[54] Doig GS, Simpson F (2005). Randomization and allocation concealment: a practical guide for researchers. J Crit Care.

[55] Bliven BD, Kaufman SE, Spertus JA (2001). Electronic collection of health-related quality of life data: validity, time benefits, and patient preference. Qual Life Res.

[56] Qualtrics. Provo, UT, USA. https://www.qualtrics.com. Accessed 31 July 2020.

[57] VERBI Software*.* MAXQDA 2020*.* Software*.* 2019. maxqda.com. Accessed 30 Mar 2020.

[58] Electronic Irish Statute Book (2018). Data Protection Act.

[59] Field A (2013). Discovering statistics using IBM SPSS statistics.

[60] Singer JD, Willett JB (2003). Applied longitudinal data analysis: modeling change and event occurrence.

[61] Little RJ, D’Agostino R, Cohen ML, Dickersin K, Scott S, Emerson MD (2012). The prevention and treatment of missing data in clinical trials. N Engl J Med.

[62] Charmaz K (2006). Constructing grounded theory: a practical guide through qualitative analysis.

[63] Department of Children and Youth Affairs (2017). Children first national guidance for the protection and welfare of children.

[64] Afzelius M, Plantin L, Östman M (2018). Families living with parental mental illness and their experiences of family interventions. J Psychiatr Ment Health Nurs.

[65] Oltedal S, Garratt A, Johannessen JO (2007). Psychiatric outpatients’ experiences with specialized health care delivery. A Norwegian national survey. J Ment Health.

[66] Romi T, Melamed S (2007). Involving the family of patients with mental illness in treatment: a model for assessment. J Fam Psychother.

[67] Van Doesum KTM, Riebschleger J, Carroll J, Grové C, Lauritzen C, Mordoch E (2016). Successful recruitment strategies for prevention programs targeting children of parents with mental health challenges: an international study. Child Youth Serv.

[68] National Institute for Health Research (2020). Restart framework: a framework for restarting NIHR research activities which have been paused due to COVID-19.

[69] Department of Health and Department of Business, Enterprise and Innovation (2020). Return to work safely protocol.

